# Asymmetric real-time PCR and multiplex melting curve analysis with TaqMan probes for detecting *PIK3CA* mutations

**DOI:** 10.1016/j.dib.2015.10.046

**Published:** 2015-11-14

**Authors:** Irina V. Botezatu, Irina O. Nechaeva, Аnna М. Stroganova, Anastasia I. Senderovich, Valentina N. Kondratova, Valery P. Shelepov, Anatoly V. Lichtenstein

**Affiliations:** N.N. Blokhin Russian Cancer Research Center, Kashirskoe Shosse 24, 115478 Moscow, Russia

**Keywords:** Mutation scanning, DNA melting analysis, TaqMan probes, PIK3CA, Multiplex analysis

## Abstract

The data in this article are related to the research article entitled “Optimization of melting analysis with TaqMan probes for detection of KRAS, NRAS, and BRAF mutations” Botezatu et al. [1]. Somatic mutations in the *PIK3CA* gene (“hot spots” in exons 9 and 20) are found in many human cancers, and their presence can determine prognosis and a treatment strategy. An effective method of mutation scanning *PIK3CA* in clinical laboratories is DNA Melting Analysis (DMA) (Vorkas et al., 2010; Simi et al., 2008) [2], [3]. It was demonstrated recently that the TaqMan probes which have been long used in Real Time PCR may also be utilized in DMA (Huang et al., 2011) [4]. After optimization of this method Botezatu et al. [1], it was used for multiplex scanning *PIK3CA* hotspot mutations in formalin-fixed paraffin-embedded (FFPE) samples from patients with colorectal and lung cancer.

**Specifications Table**

**Table:** 

Subject area	Medicine, Biology
More specific subject area	Molecular biology, Cancer Research
Type of data	Table, figure
How data was acquired	PCR, DNA melting analysis with TaqMan probes using CFX96 real-time PCR detection system (Bio-Rad Laboratories, USA)
Data format	Raw, analyzed
Experimental factors	DNA was isolated from cultured MCF-7 cells and tumor samples (colon and lung cancer).
Experimental features	Asymmetric PCR and multiplex DNA melting analysis with TaqMan probes were used for detection of mutations in *PIK3CA* exons 9 and 20.
Data source location	Moscow, Russian Federation
Data accessibility	Data is available with this article

**Value of the data**

• *PIK3CA* two primer pairs provide pseudogene-free amplification of two mutation hot spots in exons 9 and 20.
• Asymmetric real-time PCR and multiplex melting curve analysis with TaqMan probes are effective in clinical setting for evaluation of the status (wild-type or mutant) of clinically important genes.
• This cost-effective method executed in the “closed format” is simple, versatile and sensitive.

## 1. Data

The multiplex assays with TaqMan probes were carried out to amplify two regions of *PIK3CA* gene (in exons 9 and 20) and analyze the amplicons by DMA for the presence of the “hot spot” mutations (codons 542–546 and 1047–1049, respectively). This short (about 1.5 h) procedure is implemented in the “closed tube” format minimizing the sample cross-contaminations [4].

## 2. Experimental design, materials and methods

### 2.1. DNA isolation

DNA was isolated from cultured MCF-7 cells and tumor samples (colon and lung cancer) by phenol–chloroform deproteinization, and from FFPE samples by using the QIAamp DNA FFPE Tissue Kit (Qiagen) as recommended by the manufacturer. DNA concentrations were determined spectrophotometrically (Nano-Drop 1000, Thermo Scientific). DNA samples were stored at −20 °C until needed

### 2.2. Primer and probe design

Primers and probes used for *PIK3CA* melting curve analysis are presented in Table 1. Designations of the probes include the name of the amplicon (PIK9 or PIK20), fluorophore (ROX or Cy5), length and direction (sense and antisense probes are designated by subscript signs *s* and *as*, respectively). Selection of which of the two strands of the amplicon (sense or antisense) to be a target for a TaqMan probe is important, as follows from the thermodynamic calculations [5], and can affect discrimination between wild type and mutant alleles [1]. The primer set PIK9-f and PIK9-r is designed with two mismatches at the 3′-end of the reverse primer to a highly homologous (98%) chromosome 22 region to discriminate against the pseudogene and avoid false positives [2].

### 2.3. Sanger sequencing

Bi-directional analysis was performed by the fluorescent dideoxynucleotide termination method (Syntol, Moscow, Russia).

### 2.4. Asymmetric real time PCR and DNA melting analysis

Amplification reactions were carried out in 96-well plates in CFX96 Real Time PCR detection system (Bio-Rad Laboratories, Hercules, CA). Each 25-µl reaction contained 50 mM Tris–HCl, pH 8.8, 50 mМ KCl, 0.01% (v/v) Tween 20, 3 mM MgCl_2_, 0.25 мМ dNTPs, 1.25 U *Taq* polymerase, a primer pair (0.04 µМ/0.4 µM), 0.2 µM TaqMan probe (Syntol, Moscow), and 5 µl of DNA template. Real Time PCR protocols started with a denaturation step for 5 min at 95 °C, followed by 55 cycles of 95 °C for 13 s, 56 °C for 15 s, 72 °C for 20 s with fluorescence acquisition at 72 °C. After asymmetric PCR, DNA was heated at 95 °C for 1 min and incubated at 50 °C for 2 min, after which it was melted from 50 °C to 82 °C (increment 0.4 °C, dwell time 6 s, rate of heating 3.3 °C/s). The Bio-Rad CFX96 Manager software (version 1.6) was used to collect and analyze amplification and melting data from the CFX96 Real Time PCR detection system.

### 2.5. PIK3CA mutation scanning

The assays were used to analyze 19 FFPE samples from patients with colorectal and lung cancer, among which 14 were detected to harbor *PIK3CA* mutations, as confirmed by bi-directional Sanger sequencing (Table 2).

**Fig. 1:**
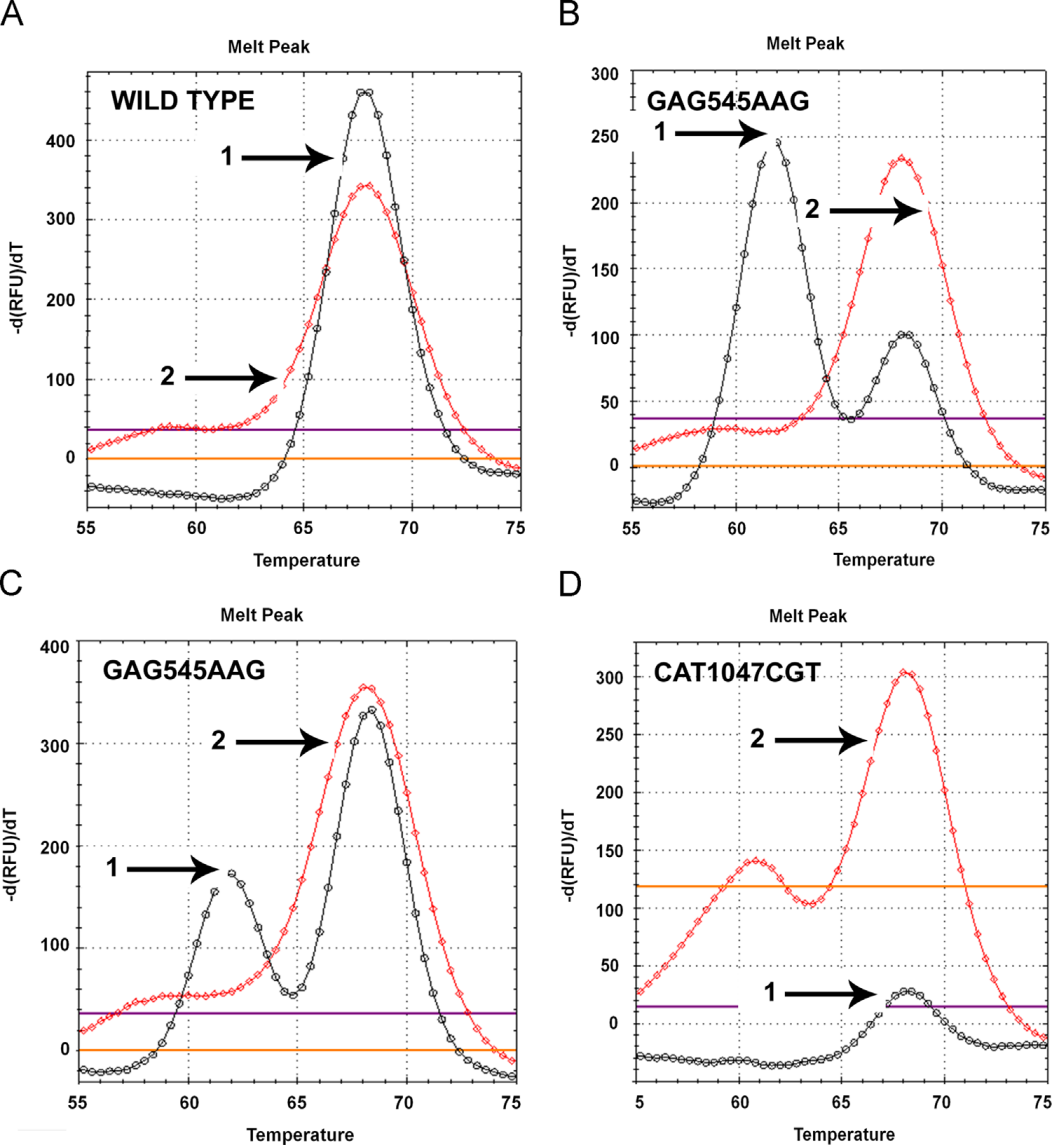
Multiplex DMA of *PIK3CA* mutations (exons 9 and 20) with PIK9-ROX(28)_as_ and PIK20-Cy5(23)_s_ probes, respectively. (A) – wild-type; (В) – MCF-7 cells (heterozygous mutation in exon 9-GAG545AAG) [2], [3]; (C)-colon cancer (mutation in exon 9); (D) – colon cancer (mutation in codon 20). Curves 1 and 2 refer to exons 9 and 20, respectively. Mutation types (determined by Sanger sequencing) are shown in figures.

The concordance rate between sequencing and DMA data was 100%. Typical DMA melting peaks for the frequent *PIK3CA* mutations are shown in Fig. 1.

## Figures and Tables

**Table 1:** Amplicons, primers and probes used for mutation scanning *PIK3CA* by DMA.

**Amplicon (exon, codons, size)**	**Primers (forward, reverse), probes (sense, antisense)**	**Sequences, sizes**
**PIK9** (exon 9, c. 542–546, 191 bp)	Primer PIK9-f	5’-GGGGAAAAATATGACAAAG (19 b)
Primer PIK9-r	5’-CATTTTAGCACTTACCTGTGAC (22 b)
Probe PIK9-ROX(28)_as_	ROX-CTTTCTCCTGCTCAGTGATTTCAGAGAG-BHQ2 (28 b)
**PIK20** (exon 20, c. 1047–1049, 194 bp)	Primer PIK20-f	5’-TGATGACATTGCATACATTC (20 bp)
Primer PIK20-r	5’-TCCAGAGTGAGCTTTCATT (19 bp)
Probe PIK20-Cy5(23)_s_	Cy5-ATGATGCACATCATGGTGGCTGG-BHQ2 (23 bp)

**Table 2:** Comparison of Sanger sequencing and DMA data for the *PIK3CA* mutations.

**Gene**		**Sanger sequencing**	**DMA**^a^
1.	GAG545AAG		+
2.	GAA542AAA		+
3.	GAG545AAG		+
4.	GAG545AAG		+
5.	GAA542AAA		+
6.		Wild Type	−
7.		GAG545AAG	+
8.		GAG545AAG	+
9.		Wild Type	−
10.		GAG545AAG	+
11.		GAG545AAG	+
12.		Wild Type	−
13.		GAG545AAG	+
14.		GAG545GGG	+
15.		Wild Type	−
16.		Wild Type	−
17.		CAT1047CGT	+
18.		CAT1047CGT	+
19.		CAT1047CGT	+

a −, wild type; +, mutation detected.
